# Universal organization of resting brain activity at the thermodynamic critical point

**DOI:** 10.3389/fnsys.2013.00042

**Published:** 2013-08-22

**Authors:** Shan Yu, Hongdian Yang, Oren Shriki, Dietmar Plenz

**Affiliations:** Section on Critical Brain Dynamics, National Institute of Mental Health, NIHBethesda, MD, USA

**Keywords:** neuronal avalanches, LFP, MEG, critical exponents, phase transition

## Abstract

Thermodynamic criticality describes emergent phenomena in a wide variety of complex systems. In the mammalian cortex, one type of complex dynamics that spontaneously emerges from neuronal interactions has been characterized as neuronal avalanches. Several aspects of neuronal avalanches such as their size and life time distributions are described by power laws with unique exponents, indicating an underlying critical branching process that governs avalanche formation. Here, we show that neuronal avalanches also reflect an organization of brain dynamics close to a thermodynamic critical point. We recorded spontaneous cortical activity in monkeys and humans at rest using high-density intracranial microelectrode arrays and magnetoencephalography, respectively. By numerically changing a control parameter equivalent to thermodynamic temperature, we observed typical critical behavior in cortical activities near the actual physiological condition, including the phase transition of an order parameter, as well as the divergence of susceptibility and specific heat. Finite-size scaling of these quantities allowed us to derive robust critical exponents highly consistent across monkey and humans that uncover a distinct, yet universal organization of brain dynamics. Our results demonstrate that normal brain dynamics at rest resides near or at criticality, which maximizes several aspects of information processing such as input sensitivity and dynamic range.

The cerebral cortex of the mammalian brain consists of tens of billions of neurons with interactions among them that exist at many scales ranging from local microcircuits, to cortical areas, and even across the entire cortex. These myriads of neuronal interactions underlie various brain functions including motion, perception, and cognition (Abeles et al., 1993; Vaadia et al., 1995; Rodriguez et al., 1999; Singer, 1999). Understanding the general principles governing these interactions and how they give rise to emergent properties of information processing is one of the most challenging questions in systems neuroscience.

For several decades, concepts and tools developed in statistical physics have addressed the collective behavior of complex systems by studying the interactions among the constituent microscopic system components. Of the many states a complex system might adopt, the critical state at thermodynamic equilibrium has been extensively studied and this state might be particularly relevant for the brain. Microscopically, the critical state represents exquisitely balanced interactions among all system components (Stanley, 1999). Macroscopically, such balanced interactions poise the system at a transition between two contrasting phases (quantified by the order parameter, *M*) and give rise to a number of non-trivial emergent properties, including the divergence of the sensitivity to external perturbations (quantified by the susceptibility, χ), and internal complexity/diversity (quantified by the specific heat, *C*; Stanley, 1987; Binney et al., 1992; Sornette, 2006). For the cortex, these quantities have intuitive meanings in terms of neuronal information processing. χ reflects the input sensitivity of the system (Newman and Barkema, 1999), *C* reflects the dynamic range of neuronal populations in representing inputs (Tkacik et al., 2009; Macke et al., 2011), and *M* measures the overall neuronal activity level. The maximization of χ and *C* achieved at criticality can thus be interpreted as optimizing input sensitivity (Houweling and Brecht, 2007; Huber et al., 2008; Shew et al., 2009) and dynamic range (Shew et al., 2009; Tkacik et al., 2009; Macke et al., 2011), respectively. At the same time, the changes of *M*, i.e., the overall activity level, may reflect state changes of the brain, such as transitions between sleep and wakefulness or between focused attention and inattention (Cohen and Maunsell, 2009; Mitchell et al., 2009; Vyazovskiy et al., 2009; Harris and Thiele, 2011; Grosmark et al., 2012).

Importantly, near the critical state, those emergent behaviors do not depend on the specific microscopic realization of a system. It has been shown that a multitude of systems can be categorized into a small number of “universality classes” based on only a few parameters, i.e., so called “critical exponents” (Stanley, 1987, 1999; Binney et al., 1992; Sornette, 2006). Within individual classes, apparently different systems follow the same quantitative rules. A major question thus arises, whether such universality of critical behavior, encountered when studying physical systems, might also include biological complex systems such as the cortex that evolved to process information.

Recent studies of neuronal avalanches strongly suggest that neuronal interactions, both at the mesoscopic scale (within tens of mm^2^ of cortical tissue; Beggs and Plenz, 2003; Petermann et al., 2009) as well as macroscopic level (across the entire cortex; Allegrini et al., 2010; Tagliazucchi et al., 2012; Palva et al., 2013; Shriki et al., 2013), may position the cortex at or near a non-equilibrium critical state in order to optimize information processing (Kinouchi and Copelli, 2006; Rämö et al., 2007; Shew et al., 2009, 2011; Yang et al., 2012). Neuronal avalanches are intermittent cortical activity cascades that spontaneously form in the normal brain. During an avalanche, spontaneous activation of one neuronal group can trigger consecutive activations of other neuronal groups within just a few milliseconds and the propagation of such activity spans both spatial and temporal domains. This propagation is well-described by a non-equilibrium critical branching process, which successfully explains some of the functional advantages of neuronal avalanches (Beggs and Plenz, 2003; Shew et al., 2009, 2011; Yang et al., 2012). However, it is currently unclear if neuronal avalanches indicate cortical dynamics close to a critical state in the equilibrium thermodynamic sense and, if so, what universality class this form of cortical activity might belong to. The current study is aimed to address these questions and their potential functional implications for the brain.

## Materials and methods

### Local field potential (LFP) recordings in monkeys

**Figure 1:**
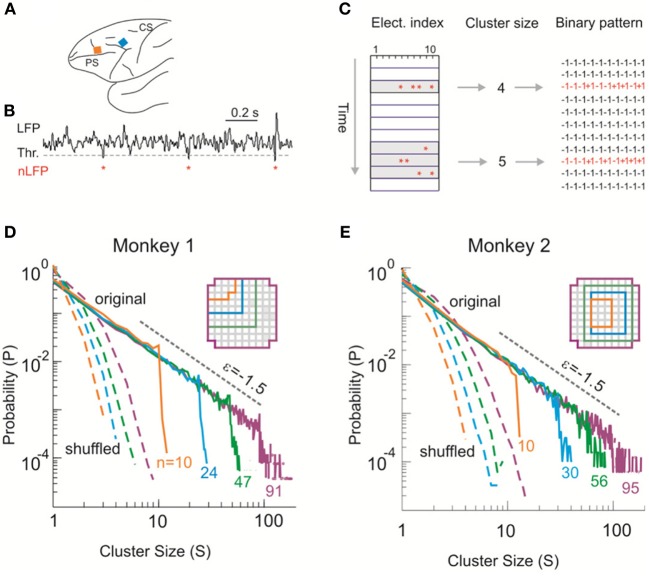
**Identifying avalanche dynamics in LFP signals. (A)** Lateral view of the macaque brain showing the position of the multi-electrode array (square, not to scale) in pre-motor (Monkey 1; blue) and prefrontal (Monkey 2; orange) cortex. PS, Principal Sulcus; CS, Central Sulcus. **(B)** Example period of continuous LFP at a single electrode. Asterisks indicate peaks of negative deflections in the LFP (nLFPs) that pass the threshold (Thr., broken line; −2.5 SD). **(C)** Identification of spatiotemporal nLFP clusters and corresponding spatial patterns. Left: nLFPs that occur in the same time bin or consecutive bins of length Δ*t* define a spatiotemporal cluster, whose size is given by its number of nLFPs (two clusters of size 4 and 5 shown; gray area). Right: Patterns represent the spatial information of clusters only. **(D**,**E)** Neuronal avalanche dynamics are identified when the sizes of activity cascades distribute according to a power-law with slope close of −1.5. Four distributions from the same original data set (solid line) using different areas (inset), i.e., number of electrodes (*n*), are superimposed. The power-law distributions vanish for shuffled data (broken lines). A theoretical power-law with slope of −1.5 is provided as guidance to the eye (gray, broken line). **(D)** is reprinted from Yu et al. (2011).

All experiments were carried out in accordance with NIH guidelines for animal use and care. The protocol was approved by the Animal Care and Use Committee of the National Institute of Mental Health. Ongoing LFP activity was recorded from two adult monkeys (*Macaca mulatta*). Multi-electrode arrays (10 × 10; 400 μm inter-electrode distance; 1 or 0.6 mm electrode length; BlackRock Microsystems) were chronically implanted in the left pre-motor (Monkey 1) or prefrontal (Monkey 2) cortex (Figure 1A). Twenty to thirty min of ongoing LFP (1–100 Hz) signals were simultaneously obtained from each electrode while the animals were sitting alert in a primate chair but not engaged in any behavioral task. For more experimental details, see Yu et al. (2011).

### Magnetoencephalography (MEG) recordings in human subjects

All experiments were carried out in accordance with NIH guidelines for human subjects. Ongoing brain activity (~30 min) was recorded from 3 healthy female participants. The sampling rate was 600 Hz, and the data were band-pass filtered between 1 and 150 Hz. The sensor array consisted of 275 axial first-order gradiometers. Two dysfunctional sensors were removed, leaving 273 sensors in the analysis. Analysis was performed directly on the axial gradiometer waveforms. For more details, see Shriki et al. (2013).

### Avalanche analysis

Negative deflections in the LFP (nLFPs) were detected by applying a threshold at −2.5 standard deviations (SDs) of the LFP fluctuations estimated for each electrode separately (Figure 1B). Such a threshold is based on the non-linear relation between nLFP amplitudes and ability of local neuronal groups to synchronize with other, spatially separated ones (Thiagarajan et al., 2010; Yu et al., 2011). The nLFP peak times were then binned using a time window, Δ*t*. Results shown are based on Δ*t* = 2 ms (Monkey 1) and 4 ms (Monkey 2) but they are similar across a wide range of Δ*t* (2–16 ms tested). Spatiotemporal clusters of nLFPs, i.e., avalanches, were defined by consecutive bins such that each bin contained at least one nLFP at any site in the selected group (Beggs and Plenz, 2003). The size of a cluster, *s*, was defined as the number of nLFPs in the cluster (Figure 1C). Similar analysis was applied to identify avalanches from the MEG recordings, for which a threshold at −3.0 SD of the MEG waveforms was used to detect significant neuronal events. The time window Δ*t* was 1.67 (1 × sampling period; subject 1) or 3.34 ms (2 × sampling period; subjects 2, 3). For more details, see Shriki et al. (2013). Avalanche patterns were obtained by collapsing all time bins within an avalanche to form a corresponding spatial pattern σ = (σ_1_, σ_2_, …,σ_*n*_), where *n* is the number of recording sites, i.e., system size, included in the analysis and σ_*i*_ = 1 if at least one nLFP occurred at site *i* and σ_*i*_ = −1 otherwise (Figure 1C).

### Using the dichotomized gaussian (DG) model for estimating pattern probabilities *P*_*i*_

The DG model is a useful tool for capturing the statistics of binary neural activity patterns (Amari et al., 2003; Macke et al., 2009, 2011; Yu et al., 2011). It applies a threshold to multivariate Gaussian variables: *y*_*i*_ = 1 when *u*_*i*_ > 0 and *y*_*i*_ = −1 otherwise, where **u** = (*u*_1_, *u*_2_, …, *u*_*n*_) ~ *N* (δ, λ), δ is the mean and λ is the covariance of the Gaussian variables. In order to match the rate, *r*, and covariance, Σ, of the observed binary variables, i.e., avalanche patterns, δ and λ need to be adjusted according to δ_*i*_ = Φ ^−1^(*r*_*i*_) and λ_*ij*_ as the solution for Σ_*ij*_ = Φ_2_ (δ_*i*_, δ_*j*_, λ_*ij*_) – Φ(δ_*i*_) Φ (δ_*j*_), where Φ and Φ^−1^ are the cumulative probability function of a Gaussian distribution (Φ for 1-dimensional and Φ_2_ for 2-dimensional) and its inverse function, respectively. An implementation of the model in MATLAB can be found in Macke et al. (2009). The pattern probabilities for the DG model were obtained by calculating the cumulative distribution of multivariate Gaussians (MATLAB function *mvncdf*).

### Fitting a power-law to the size distribution

The exponent of the best fitting power-law, was estimated by minimizing the Kolmogorov–Smirnov (KS) distance between the empirical distribution and a power-law distribution (Klaus et al., 2011). The KS distance (*D*_KS_) was defined as

(1) $$D_{\text{KS}}=\underset{s}{\max}|CDF_{\text{emp}}\left(s\right)-CDF_{\text{power}-\text{law}}\left(s\right)|,$$

where *s* is the pattern size and *CDF*_emp_ and *CDF*_power−law_ represent the cumulative distribution function for the empirical size distribution and the power-law function used for fitting, respectively.

### Inferring *p*_*i*_ for different values of T

To predict the pattern probabilities *p*_*i*_ for different values of the fictitious temperature, *T*, it is useful to express the state probability as a function of interactions that occur at different orders (Nakahara and Amari, 2002; Amari et al., 2003). Let the pattern probability be *p*(σ), where σ = (σ_1_, σ_2_, …, σ_*n*_) and σ_*n*_ = {1, −1}, representing the states of individual components. Generally, we can write *p*(σ), using the full log-linear expansion, as

(2) $$p\left(\sigma \right)=\frac{1}{Z}\exp\left(\sum_{i}\theta_{i}\sigma_{i}+\sum_{\left(i<j\right)}\theta_{ij}\sigma_{i}\sigma_{j}\text{​​}+\sum_{\left(i<j<k\right)}\theta_{ijk}\sigma_{i}\sigma_{j}\sigma_{k}+\;\cdots \text{​}\right),$$

where *Z* is the normalization factor and θ characterizes different orders of interactions. The full log-linear expansion and its lower-order approximations have been widely used in characterizing neuronal interactions (Schneidman et al., 2006; Yu et al., 2008; Ohiorhenuan et al., 2010).

Next, we define θ = θ^0^/*T*, where θ^0^ represent the intrinsic interaction strength that does not depend on *T*. If we denote $E\left(\sigma \right)=-\left(\sum_{i}\theta_{i}^{0}\sigma_{i}+\sum_{\left(i<j\right)}\theta_{ij}^{0}\sigma_{i}\sigma_{j}+\sum_{\left(i<j<k\right)}\theta_{ijk}^{0}\sigma_{i}\sigma_{j}\sigma_{k}+\;\cdots \right)$, Equation 2 can be rewritten as

(3) $$p\left(\sigma \right)=\frac{1}{Z}\exp\left(\frac{-E\left(\sigma \right)}{T}\right).$$

We can then use the single histogram method (Ferrenberg and Swendsen, 1988; Newman and Barkema, 1999) to infer *p*_*i*_ for different *T*, an approach that was used for modeling natural image statistics (Stephens et al., 2013) and was also recently introduced to neuroscience (Tkacik et al., 2009). Specifically, if *p*_*i*_ denotes the probability of any given pattern *i* and *E*_*i*_ the corresponding *E*, Equation 3 changes to

(4) $$p_{i}=\frac{1}{Z}e^{-E_{i}/T}$$

Setting *T* = 1 for the original recording, Equation 4 can be expressed as

(5) $$p_{i}\left(1\right)=\frac{1}{Z\left(1\right)}e^{-E_{i}},$$

which enables us to compute *p*_*i*_ for different *T* as

(6) $$p_{i}\left(T\right)=\frac{1}{Z}e^{\frac{-Ei}{T}}=\frac{1}{Z}{\left[Z\left(1\right)p_{i}\left(1\right)\right]}^{\frac{1}{T}}=\frac{Z{\left(1\right)}^{1/T}}{Z}p_{i}{\left(1\right)}^{1/T}$$

The normalization factor is determined by considering ∑*p*_*i*_(*T*) = 1.

### Computing the specific heat, susceptibility, and order parameter

The specific heat, *C*, is:

(7) $$C=\frac{1}{n}\frac{\partial U}{\partial T}=\frac{\langle E_{i}^{2}\rangle -{\langle E_{i}\rangle }^{2}}{nT^{2}},$$

where *n* is system size, *U* ≡ 〈*E*_*i*_〉 = ∑*p*_*i*_*E*_*i*_ and *E*_*i*_ can be calculated according to Equation 4. Given *n* and *T*, *C* reflects the variance of log (*p*_*i*_), a useful metric for quantifying the capacity of the system to represent information (Tkacik et al., 2009; Macke et al., 2011).

The order parameter, *M*, is defined as:

(8) $$M=\frac{1}{n}\sum_{i=1}^{2^{n}}p_{i}m_{i},$$

where *m*_*i*_ = ∑^*n*^_*j* = 1_σ^*i*^_*j*_. σ^*i*^ indicates that the value of σ is taken from the *i*^th^ pattern. *M* has a very intuitive meaning for a cortical system—it reflects the overall activity level of the system.

Finally, the susceptibility χ is a measure of the sensitivity of the system to small external perturbations. χ is defined as the change rate of *M* when a small external field *H* is applied:

(9) $$\chi =\frac{\partial M}{\partial H}|{\;}_{H=0}=\frac{\langle m_{i}^{2}\rangle -{\langle m_{i}\rangle }^{2}}{nT}$$

The field *H* exerts its effect by changing the preference of the units to be active or not, i.e., their likeliness to be involved in an avalanche. Specifically, applying *H* is equivalent to adding a term of *H*Σσ_*i*_ to the Hamitonian (*E*). For cortical dynamics, *H* can be thought as an approximation of a local perturbation, e.g., making a single or small group of neurons to fire [analog to flipping a single spin in a model; see Newman and Barkema (1999) and/or a weak common input from, e.g., distant cortical areas or sub-cortical brain structures].

### Finite size scaling (FSS) analysis

At the thermodynamic limit (*n* → ∞), a critical system can be identified by power-law behaviors of its macroscopic quantities, including the correlation length ξ (a characteristic distance beyond which correlations diminish), specific heat *C*, magnetization *M* and susceptibility χ. These quantities follow a power-law relation as a control parameter, such as the thermodynamic temperature *T*, approaches a critical value *T*_*c*_, with specific critical exponents ν, α, β, and γ, respectively:

(10) $$\xi ∼{\left|t\right|}^{-\nu }$$

(11) $$C\sim {\left|t\right|}^{-\alpha }$$

(12) $$M\sim {\left|t\right|}^{-\beta }$$

(13) $$\chi \sim {\left|t\right|}^{-\gamma }$$

where *t* = (*T* − *T*_*c*_)/*T*_*c*_. In principle, one could directly measure these relations to determine whether and when the system will be critical, i.e., to determine *T*_*c*_, and, at the same time, estimate all critical exponents.

The complication comes with the fact that real systems are finite in size. This so called “finite size effect” causes the system's behavior to deviate from the thermodynamic limit. A standard procedure in statistical physics to solve this problem is Finite Size Scaling (FSS; Binney et al., 1992; Newman and Barkema, 1999). By analyzing the behavior of systems with different sizes, FSS extrapolates the behavior for the thermodynamic limit and to estimate *T*_*c*_ and critical exponents. Briefly, we can choose a unique set of critical exponents to scale Equations 10–13 with different linear sizes of the system $L=\sqrt[{d}]{n}$, where *d* is the dimensionality, and then collapse the curves obtained for all sizes. Specifically, *t* needs to be scaled by *L*^1/ν^, whereas *C*, *M*, and χ are scaled by *L*^−α/ν^, *L*^β/ν^, and *L*^−γ/ν^, respectively. The critical exponents (ν, α, β, and γ) and *T*_*c*_ that achieve the collapse are equivalent to those expected for a measurement made at the thermodynamic limit (see Appendix for detailed derivation). We identified the best collapse by minimizing the distance among all functions with different sizes using numerical optimization (MATLAB function *fminsearch*). Initial conditions for optimization were systematically changed according to a grid search method within a large parameter space and the resulting values for exponents were stable. These values were also stable for different values of *T* to perform FSS. Results reported were based on *T* = 0.5 − 2.5.

### Measuring goodness of collapse

For different system sizes *i*, the dependency of a system parameter, e.g., susceptibility χ_*i*_, on *T* was obtained. To quantify how well such a series of functions can be collapsed by FSS, we compared the “closeness” of them before (without scaling) and after the collapse (the best results achieved by numerical optimization). Specifically, the goodness of collapse (*GC*) is indicated by the ratio of mean squared deviation (MSD) after and before the collapse, i.e., *GC* = MSD^after^/MSD^before^. Formally, $\text{MSD}={\langle {\langle {\left(\chi_{i}-\bar{\chi }\right)}^{2}\rangle }_{T}\rangle }_{i}$, where $\bar{\chi }$ is the point-wise average over all system sizes, 〈〉_*T*_ indicates the average across the range of *T* and 〈〉_*i*_ indicates the average across system sizes. Smaller *GC* indicates better goodness of collapse.

## Results

### Avalanche dynamics at the mesoscopic scale

We first investigated neuronal avalanches at the mesoscopic scale (Beggs and Plenz, 2003; Petermann et al., 2009; Hahn et al., 2010; Ribeiro et al., 2010; Yu et al., 2011). Ongoing neuronal activity in two monkeys was recorded with 10 × 10 high-density micro-electrode arrays chronically implanted in superficial layers of cortex (Figure 1A). Significant negative local field potential deflections (nLFPs), which indicate synchronized activity of local neuronal populations (Petermann et al., 2009; Yu et al., 2011), were detected using an amplitude threshold of –2.5 SDs of the LFP calculated for each electrode (Figure 1B). A spatiotemporal nLFP cluster was identified if nLFPs on the multielectrode array occurred within the same or consecutive time bins of width Δ*t* (Figure 1C). Importantly, the cluster size s, defined as the number of nLFPs in a cluster, distributed according to a power-law with an exponent close to −1.5. Moreover, the distribution exhibited scale-free behavior, i.e., the power-law and its slope were stable for different system size *n*, whereas the cut-off changed systematically with *n* (Figures 1D,E). This power-law demonstrates that ongoing cortical activity at rest in awake monkeys organizes as neuronal avalanches (Beggs and Plenz, 2003; Petermann et al., 2009). It indicates the presence of significant correlations in neuronal activity among cortical sites and, accordingly, is destroyed when the times of nLFPs are shuffled randomly (Figures 1D,E, broken lines).

### Characterization of the critical behavior

Next we investigated whether neuronal avalanches reflect a cortical state close to criticality in the sense of a thermodynamical equilibrium. Our approach is based on a method similar to Monte Carlo simulations (Newman and Barkema, 1999). First, we estimated the probability *p*_*i*_ of individual configurations in the system based on actual recordings. For an equilibrium system, those probabilities would give a complete characterization of the system's behavior. Then, we infer the changes of *p*_*i*_ with the change of a control parameter, *T*, which is considered to be equivalent to thermodynamic temperature. Finally, we compute various macroscopic properties including susceptibility, specific heat, and an order parameter, as a function of *T* to judge if the actual *T* (the one associated with the original recording) is close to the critical point.

**Figure 2:**
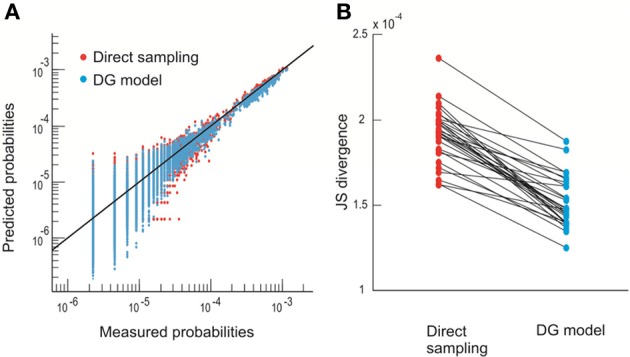
**The DG model predicts state probability more accurately than direct sampling. (A)** Observed probability *p*_*i*_ (thirty 10–electrode sub-groups) is plotted against the prediction made by direct sampling and the DG model. Solid line indicates equality. The comparison is based on 2-fold cross-validation (Yu et al., 2011). **(B)** JS divergence (Yu et al., 2011) between the observed and predicted probabilities of spatial avalanche patterns for the same thirty 10–electrode groups shown in **(A)**. Linked dots are the results obtained by direct sampling and the DG model for the same group. The DG model has significantly smaller JS divergence (21% reduction, *p* < 10^-5^, paired-sample signed rank test).

More specifically, we define the configurations or states of the system by the spatial avalanche patterns, obtained by collapsing the spatiotemporal avalanche patterns along the temporal domain. This mapping ignores the internal temporal structure of individual avalanches. Each avalanche is originally represented by an *n* by *m* activity matrix, where *n* is the number of electrodes and *m* is the temporal duration of the avalanche. The activity matrix is then turned into an *n*-component binary vector where an electrode is set to 1 if it participates at least once in the avalanche and to −1 otherwise [Figure 1C, see also Methods and Yu et al. (2011)]. The finite duration of the recording limits the direct estimation of pattern probabilities *p*_*i*_ to *n* ~ 10. Therefore, in order to estimate *p*_*i*_ for larger *n*, we take advantage of a parametric model, the Dichotomized Gaussian (DG) model (Amari et al., 2003; Macke et al., 2009, 2011; Yu et al., 2011), which considers only the observed first-order (event rate) and second-order (pair-wise correlations) statistics. This model estimates *p*_*i*_ of avalanche patterns more accurately than directly measuring it from the limited data [Figure 2; see also Yu et al. (2011)]. Due to the exponential increase in possible configurations with increasing *n*, we restrict the calculation of *p*_*i*_ to *n* = 20. In total, we analyzed four 20-electrode sub-groups recorded from each of the two monkeys.

**Figure 3:**
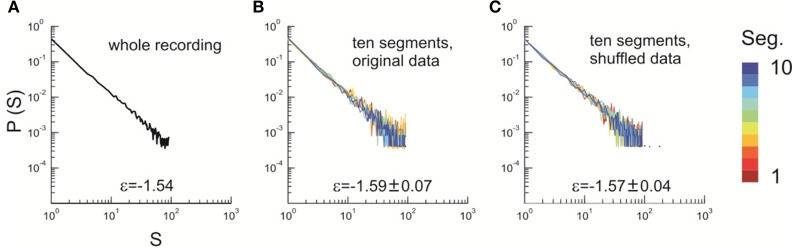
**Stability of the power law size distribution during the recording. (A)** Avalanche pattern size distribution of the whole recording (30 min) plotted in a double-logarithmic scale. ε, exponent of the best fitting power law to the distribution. Avalanche pattern was identified based on the activities recorded in the whole array (91 channels, Monkey 1). **(B)** The full dataset as analyzed in **(A)** was split into 10 consecutive, non-overlapping segments, each of which lasted for 3 min. Avalanche pattern size distributions were calculated for individual segments and plotted (color coded). **(C)** The original dataset as analyzed in **(A)** was shuffled in time (i.e., the sequence of activities was randomized) to eliminate temporal dependencies and split into ten consecutive, equal-sized segments. Avalanche pattern size distributions were calculated for individual segments and plotted (color coded). In **(B)** and **(C)**, ε is represented as mean ± s.d. (across all segments).

**Figure 4:**
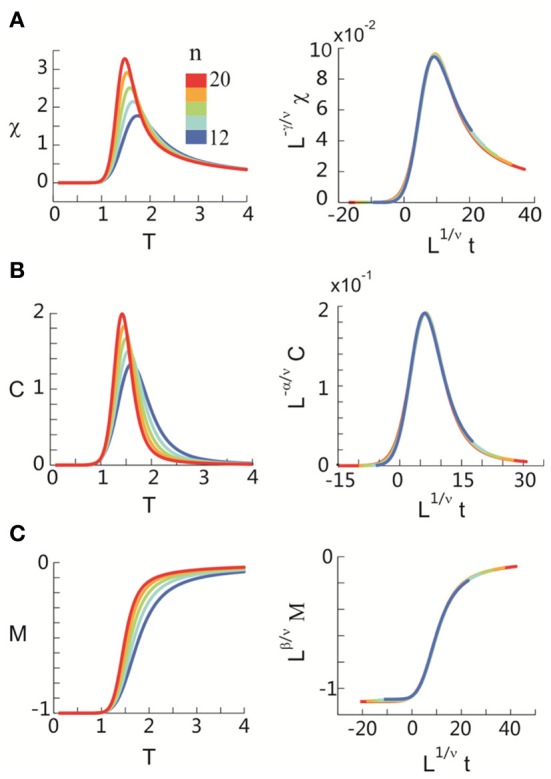
**Critical behavior in susceptibility, specific heat, and order parameter observed for neuronal avalanches at the mesoscopic level, i.e., recorded by LFPs.** Susceptibility **(A)**, specific heat **(B)**, and order parameter **(C)** are plotted as a function of *T* for system size *n* = 12–20 (color code). Left: Original non-scaled functions. Right: Corresponding collapse using FSS analysis. Scaled quantities plotted as a function of *t* = (*T* − *T*_*c*_)/*T*_*c*_, $L=\sqrt[{d}]{n}$, where *d* is the dimensionality of the system. Critical exponents: α, β, γ, and ν. We note that the peaks for the scaled variables χ and *C* are not expected to be at the location of *L*^1/ν^*t* = 0.

**Figure A1:**
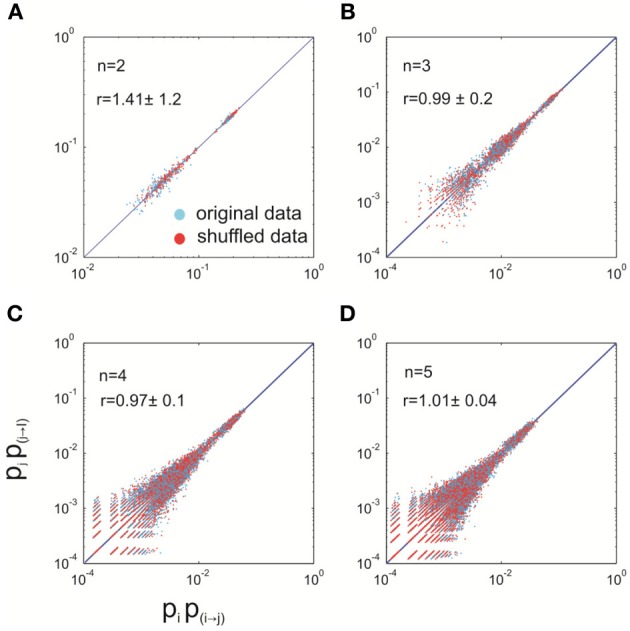
**Detailed balance approximately holds for the data with quiescent periods removed.** For differently sized systems (*n* = 2–5), empirically measured *p*_*i*_*p*_*i*→*j*_ is plotted against *p*_*j*_*p*_*j*→*i*_ for both the original data (*blue*) and shuffled data (*red*). For every size, 100 different systems (i.e., different combinations of electrodes) were analyzed. The solid lines represent equality. *r* is a measure of the distance from the equality, relative to that of the shuffled data (see Appendix text A for details). It is represented as mean ±s.d. (across 100 systems). **(A–D)**, system size equals 2, 3, 4, and 5, respectively.

After obtaining *p*_*i*_ for the condition in which the actual recording was taken, we introduce a control parameter *T*, which changes both the likelihood of a given site to participate in an avalanche and the correlation among activities between different sites (Binney et al., 1992; Newman and Barkema, 1999). *T* is similar to the thermodynamic temperature and allows us to systematically estimate the system's behavior for conditions different from the recorded, physiological condition. To infer *p*_*i*_ for different *T*, we use the single histogram method (Ferrenberg and Swendsen, 1988; Newman and Barkema, 1999), which accurately predicts behavior of equilibrium system for different values of the control parameter. We note that the equilibrium assumption for the data is supported by the stable size distribution of avalanches over time (Figure 3) and the demonstration of detailed balance (Figure A1; see Appendix for more details). If we set *T* at which the actual recording was taken to be 1, it can be shown that, $p_{i}(T)=\frac{1}{Z}p_{i}{\left(1\right)}^{1/T}$ where *p*_*i*_(*T*) is the state probability with the thermodynamic temperature *T* and *Z* is a normalization factor (Methods). After obtaining *p*_*i*_ for a wide range of *T*, we use finite size scaling (FSS) analysis (Newman and Barkema, 1999) to investigate whether the avalanche state (*T* = 1) is close to a thermodynamic critical point, i.e., if the critical “temperature” *T*_*c*_ ≈ 1. We first analyzed the thermodynamic quantities χ, C, and M as functions of *T* for different system sizes (*n* = 12 − 20; Figure 4). Those functions measured for different *n* will be scaled according to a unique set of *T*_*c*_ and critical exponents to test if they can be collapsed. Specifically, *T* needs to be scaled by *L*^1/ν^(*T* − *T*_*c*_)/*T*_*c*_, where $L=\sqrt[{d}]{n}$ and *d* is the dimensionality of the system. χ, *C*, and *M* need to be scaled by *L*^−α/ν^, *L*^β/ν^, and *L*^−γ/ν^, respectively. Achieving such a collapse implies that, at the thermodynamic limit, the system has a critical point at *T*_*c*_, which is characterized by the divergence of χ and *C* and the phase transition of *M*. To illustrate this, we consider the collapse of χ, which implies that, at *T*_*c*_, the scaled quantity of χ, i.e., *L*^−γ/ν^χ, is a constant. When *n* → ∞, *L*^−γ/ν^ = *n*^−γ/ν*d*^ → 0 because γ/ν*d* > 0 (see below). Therefore, a finite product of *L*^−γ/ν^ and χ implies χ → ∞. We find an excellent collapse up to *n* = 20 (Figure 4). Importantly, the values of *T*_*c*_ estimated by the FSS method are close to 1 (Table 1), suggesting that ensembles of neuronal avalanches are organized at the vicinity of a thermodynamic critical point. In addition to *T*_*c*_, FSS also estimates the critical exponents, including ν, α, β, and γ. They characterize how χ, *C*, and *M* change as a function of *T* at the thermodynamic limit. We find that ν ≈ (0.8 – 0.9)/*d*, α ≈ 0.7, β close to 0 and γ close to 1. These results are consistent across the datasets obtained from two monkeys (Table 1).

**Table 1:** **Critical temperature *T*_*c*_ and critical exponents ν*d*, α, β, and γ estimated using finite size scaling analysis (FSS) for eight 20-eletrode sub-groups in two monkeys (M1, M2) and six 20-sensor sub-groups in three human subjects (H1–H3)**.

**Subject**	**Group**	***T*_*c*_ (χ)**	ν**d (χ)**	γ	***T*_*c*_*(C)***	ν***d (C)***	α	***T*_*c*_ (*M*)**	ν ***d* (*M*)**	β
M1	A	1.13	0.88	1.04	1.15	0.92	0.72	1.16	0.84	−0.028
	B	1.12	0.86	1.00	1.14	0.90	0.72	1.14	0.84	−0.021
	C	1.12	0.86	0.98	1.14	0.88	0.72	1.13	0.84	0.001
	D	1.12	0.86	1.02	1.15	0.88	0.73	1.16	0.80	−0.03
M2	A	1.10	0.82	1.05	1.14	0.84	0.71	1.16	0.76	−0.03
	B	1.11	0.90	1.10	1.13	0.96	0.71	1.13	0.84	0.001
	C	1.10	0.84	1.06	1.14	0.84	0.71	1.12	0.78	0.001
	D	1.11	0.82	1.05	1.15	0.86	0.72	1.13	0.78	0.000
H1	A	1.16	0.84	1.20	1.22	0.86	0.67	1.20	0.74	0.0006
	B	1.20	1.04	1.18	1.23	1.06	0.64	1.24	0.96	−0.02
H2	A	1.17	0.82	1.21	1.22	0.84	0.68	1.20	0.74	−0.0007
	B	1.18	0.98	1.17	1.22	1.00	0.66	1.20	0.92	−0.0003
H3	A	1.14	0.82	1.09	1.17	0.86	0.67	1.16	0.78	0.0007
	B	1.18	0.98	1.02	1.20	1.00	0.65	1.17	0.98	0.0001

*Arguments in brackets indicate that T_*c*_ and νd were estimated by applying FSS to susceptibility χ, specific heat C and order parameter M, respectively*.

### Avalanche dynamics at the macroscopic scale

**Figure 5:**
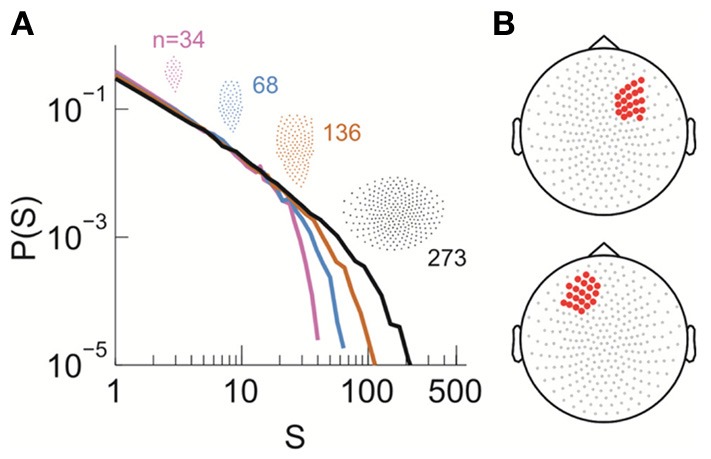
**Power law size distribution of neuronal avalanches recorded with MEG for the human brain at resting state. (A)** Neuronal avalanche dynamics are identified when the sizes (*s*) of all clusters distribute according to a power law with slope close of −1.5 (the results for subject 2 are shown here). Four distributions from the same original data set using different areas (insets), i.e., number of MEG sensors (*n*), are superimposed. **(B)** The whole array of sensors (gray dots) and two sub-groups of sensors that were used for finite-scaling analysis (red dots) are illustrated. Top, sub-group **(A)**; bottom, sub-group **(B)**. The sub-groups were identical across all three subjects.

**Figure 6:**
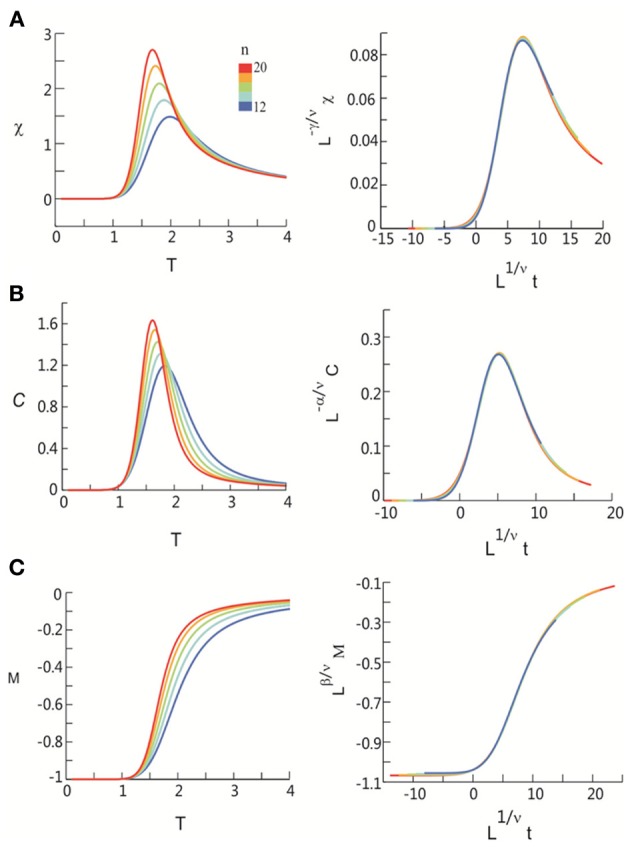
**Critical behavior in susceptibility, specific heat, and order parameter observed for neuronal avalanches in the human brain at macroscopic level, i.e., recorded with MEG.** Susceptibility **(A)**, specific heat **(B)**, and order parameter **(C)** are plotted as a function of *T* for system size *n* = 12–20 (color code). Left: Original non-scaled functions. Right: Corresponding collapse using FSS analysis. Scaled quantities are plotted as functions of “reduced temperature,” *t* = (*T* − *T*_*c*_)/*T*_*c*_, $L=\sqrt[{d}]{n}$, where *d* is the dimensionality of the system. Critical exponents: α, β, γ, and ν.

**Figure 7:**
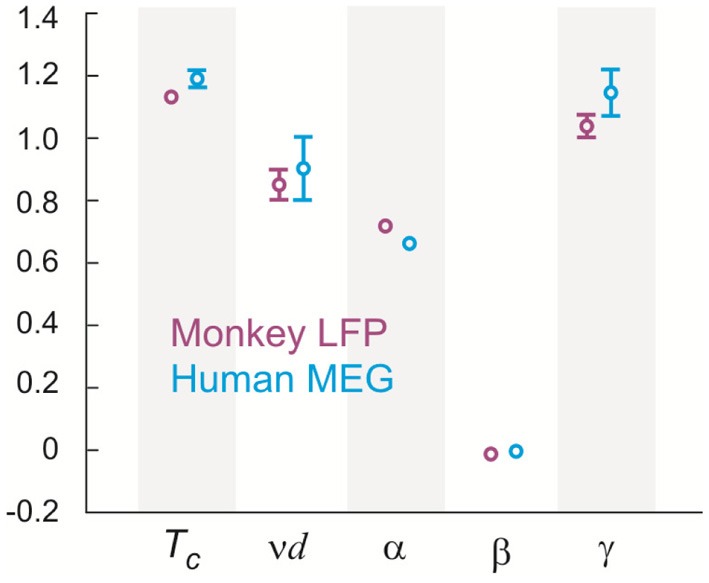
***T*_*c*_ and critical exponents α, β, γ, and ν estimated using finite size scaling analysis in two monkeys and three human subjects.** Four (two) different 20-electrode/sensor sub-groups were analyzed for each monkey (human) dataset resulting in the sample size of 8 (6). Values are mean (center circle) ± s.d. (error bars omitted for s.d. smaller than center circle).

Seeking to extrapolate from these results, we applied the FSS analysis to neural dynamics manifested at the macroscopic scale—the whole human brain—measured by MEG. In Figure 5, we show that ongoing neuronal activity in human MEG reflects neuronal avalanches, which reconfirmed our recent finding (Shriki et al., 2013). Despite the dramatically different spatial scales between the LFP and MEG signals from monkeys and humans (>10,000-fold difference in recording areas), we found strikingly similar behavior for the activity measured across the entire human cortex when the control parameter, *T*, and system size, *n*, change (Figure 6). Again, FSS analysis suggests that *T*_*c*_ ≈ 1 for the macroscopic system (Table 1). The results were consistent across different human subjects and, importantly, both *T*_*c*_ and the critical exponents of MEG recordings are very similar to those obtained from the LFP recordings (Figure 7). Such similarity, in terms of both the scaling behavior, i.e., collapse of curves, and critical exponents, strongly suggests a universal organization that underlies neuronal interactions at various spatial scales.

### Validating the FSS method through a simple model

**Figure 8:**
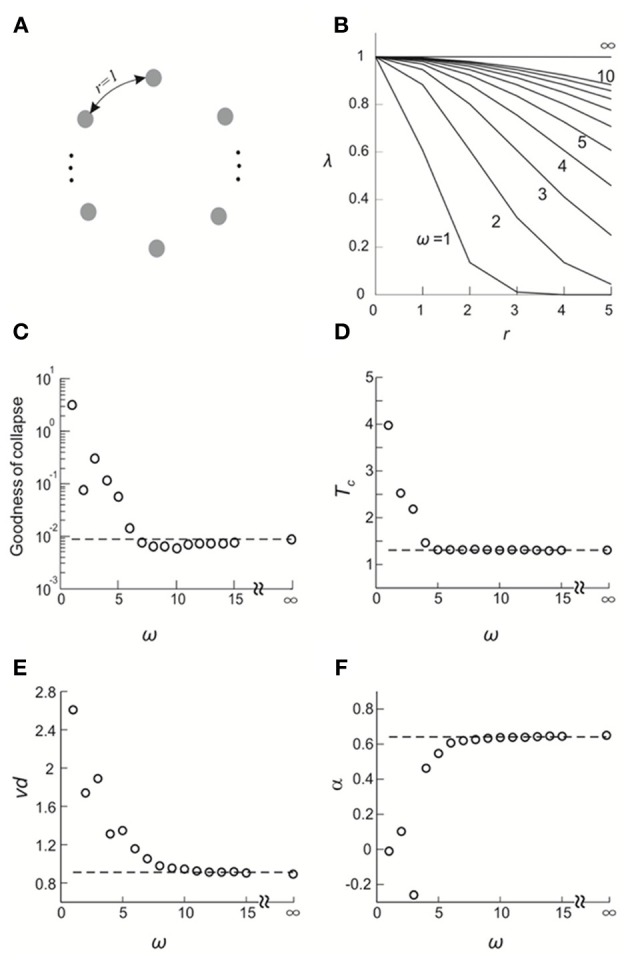
**Validating the FSS method by a simple model. (A)** All elements are configured in a ring and the distance between any adjacent elements is 1. **(B)** the covariance of the hidden variables in the DG model, λ, is plotted as a function of the distance, *r*, that separates corresponding elements for different choices of the standard deviation of a Gaussian function, ω. **(C–F)** Goodness of collapse, *T*_*c*_ and critical exponents measured for various systems are plotted against ω (open circles). In all systems, λ_max_ and mean event rate were set such that when ω = ∞, the average covariance and the event rate match what we empirically observed for Monkey 1. Corresponding results obtained from actual data for Monkey 1 (averaged across four sub-groups) are shown for comparison (broken lines).

Next, we investigated a simple and understandable model, and exemplified the sensitivity of FSS analysis to distinguish critical from non-critical system dynamics. To this end, we used the DG model in which all elements were embedded in a ring configuration. Each element had a well-defined “distance” to every other element (Figure 8A). We set the covariance of hidden variables (Methods) *i* and *j*, λ_*ij*_, as a Gaussian function of the distance *r*_*ij*_ between them: $\lambda_{ij}=\lambda_{\max}\exp\left[-\frac{1}{2}{\left(\frac{r_{ij}}{\omega }\right)}^{2}\right]$, where λ_max_ is the maximal covariance and ω is the SD of the Gaussian function. For the limit of ω → ∞, all λ_*ij*_ become identical and criticality is ensured (Macke et al., 2011). Conversely, decreasing ω to 0 drives the system to an independent state (Figure 8B).

We applied the FSS method to this system. To facilitate the analysis, system sizes were set to be *n* = 6–10. In Figures 8C–F, we plot the goodness of collapse, estimation of *T*_*c*_, and critical exponents as a function of ω. We found that for this model, the deviation from the critical state (ω = ∞) is detectable for ω <7~8. Given that all *r* ≤ 5, we consider the sensitivity of the FSS for detecting deviations from criticality as satisfactory. We note that with increasing system sizes in the analysis, even higher sensitivity will be achieved. We also compared these results with real data (*n* = 6–10) and found that the actual results we obtained for cortical activities are very close to a true critical state (Figures 8C–F), further supporting the previous results that neuronal avalanches represent a cortical state close to thermodynamic criticality.

### Correlation structure in neuronal avalanche dynamics

**Figure 9:**
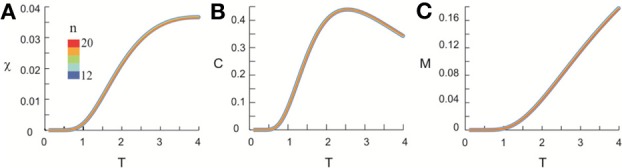
**Shuffled data does not exhibit scaling behavior.** Original data was the same as shown in Figure 4. At *T* = 1, we calculated the individual pattern probabilities based on independent Poisson processes to generate nLFPs with the same empirically measured rate for each cortical site. Using the same method applied to original data, we calculate χ, *C*, and *M* as functions of *T*. In contrast to the original data, the curves for systems of different sizes are almost identical for χ **(A)**, *C* **(B),** and *M* **(C)**. For visual clarity, curves with different sizes have different widths.

**Figure 10:**
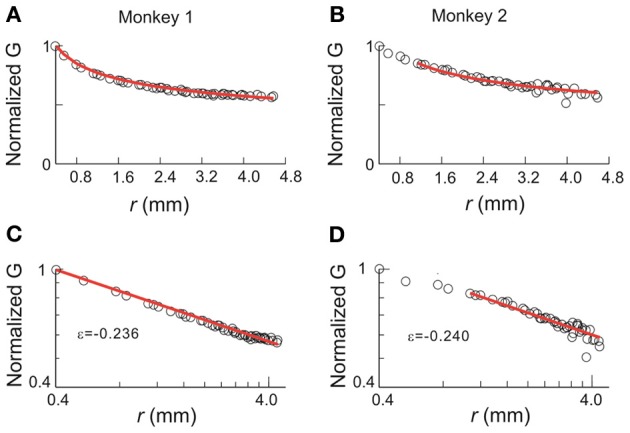
**Correlation function for avalanche activities.** Pair-wise covariance, *G*, of nLFP activities is plotted against the physical distance between the corresponding recording sites. **(A,B)** Linear coordinates. **(C,D)** Double-logarithmic coordinates. *G* is normalized by the value of the *G*(0.4), i.e., the covariance with the nearest neighbor. In all panels, the data are represented by circles and red lines indicate the best power law fit. The range of power-law fitting is either all possible distances (monkey 1) or *r* > 1 mm (monkey 2). ε, the exponent of the best fitting power law.

The results based on this simple model also provide testable predictions for the empirical data. First, if we remove all correlations in activities between cortical sites, the critical behavior observed in the original data should be abolished. To test this prediction, we used independent Poisson processes to generate nLFPs at the empirically measured rate for each cortical site. χ, *C*, and *M* were then calculated as a function of *T* and *n* in the same way as for the original data. As expected, all three quantities did not depend on system size anymore and thus did not show any scaling behavior (Figure 9). Another important prediction is that the original data should contain long-range spatial correlations. In Figure 10, we plot the correlation *G*, defined as *G*_*ij*_ = 〈σ_*i*_σ_*j*_〉 − 〈σ_*i*_〉〈σ_*j*_〉, as a function of the Euclidian distance *r* between sites *i* and *j* in both linear and log-log coordinates. We found that the correlation slowly decreases with increase in distance and that the rate of decay further decelerates at larger distance. As a result, for an increase in distance by one order of magnitude, the correlation decreases by less than 50% (Figures 10A,B), demonstrating that fluctuations in activity between very distant cortical sites are still correlated. For critical systems, theory predicts that the decay in spatial correlation should be a power law function with an exponent close to zero, which ensures the existence of long-range correlations (Binney et al., 1992). In line with theory, the spatial correlations in monkey 1 and those with distance >1 mm in monkey 2 exhibit a linear tendency in log-log coordinates, with exponents of −0.24 ± 0.05 (Figures 10C,D). The 10 × 10 recording array with interelectrode distance of 0.4 mm limits our investigation of the spatial correlation function to roughly one order of magnitude from 0.4 to 4.5 mm of distance. On the other hand, 4.5 mm already captures a relatively large distance within one cortical area of a macaque's brain. A more definitive conclusion about whether a power law is a good approximation awaits future studies with the capability to record from a much wider spatial extent. It is interesting that the data and the model with ω = ∞ share the same set of critical exponents (Figures 8E,F), despite their differences in correlation structure. Whereas *G* was constant in the model (for ω = ∞), it changed systematically as a function of *r* in the data. Consequently, all patterns with the same size were equally probable in the model (Macke et al., 2011), whereas these probabilities differed in the data by up to 2 orders of magnitude. Therefore, the fact that the model and the data share the same set of exponents is non-trivial, suggesting that they belong to the same universality class.

### Relation between the power-law size distribution and thermodynamic criticality

**Figure 11:**
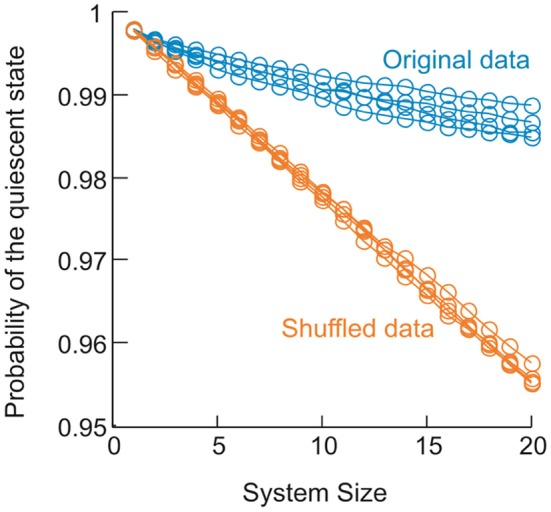
**Change in the probability of the quiescent state as a function of system size in the data.** For 4 sub-groups analyzed in monkey 1, probability of the quiescent state measured for the original data (blue) is plotted as a function of systems size (from 1 to 20). Probability of the quiescent state measured for corresponding shuffled data (orange) is plotted for comparison. Shuffled data were obtained by randomizing the activity sequence for individual electrodes, which eliminates the correlation among different electrodes but preserves the probability of being active for all electrodes.

**Figure 12:**
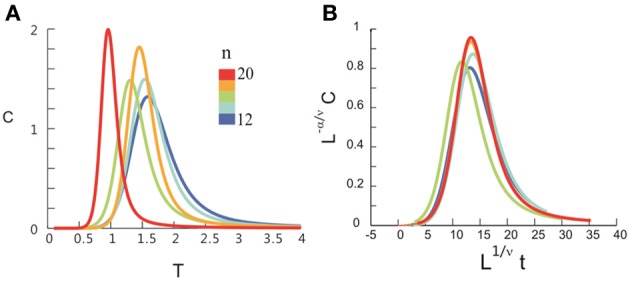
**Dissociation between the scaling/collapse and the power-law size distribution.** Pattern probabilities of the original data (as shown in Figure 4) were modified so that the probability for the quiescent state, *p*_0_, was set randomly from a uniform distribution (0, 1) while the probabilities for all other states were renormalized, i.e., *p*_*i*_ = *p*_*i*_/(1 − *p*_0_). Therefore, the power-law size distribution was preserved. **(A)**, Specific heat, *C*, is plotted as a function of *T* for system size *n* = 12 – 20 (color coded). **(B)** No collapse can be achieved.

**Figure 13:**
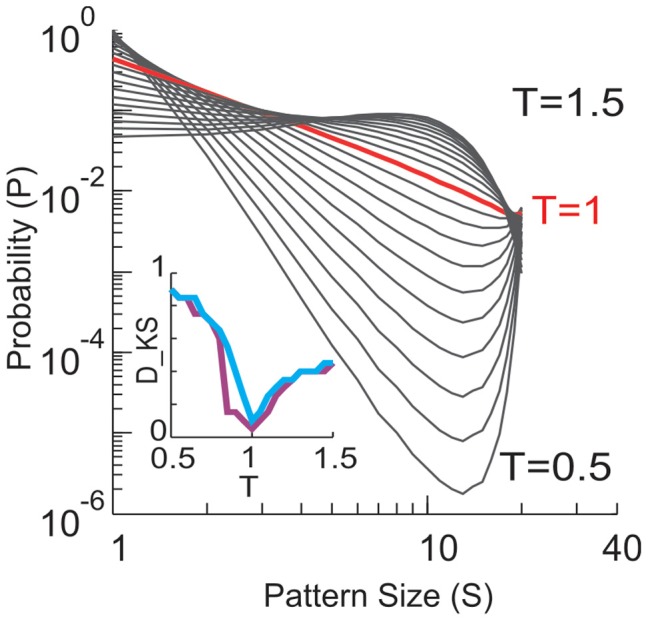
**Size distributions of avalanche patterns computed for one 20-electrode sub-group (taken from data set in Figure 1D) for different *T* and plotted in double logarithmic coordinates.** *T* changes from 0.5 to 1.5 with a step of 0.05. Distribution at *T* = 1 is marked by red. Inset: Kolmogorov–Smirnov distance (*D*_KS_, a goodness-of-fit measure) between the actual pattern size distributions and best fitting power law (purple) or power law with slope −1.5 (blue) is minimized for *T* ≈ 1.

The equilibrium critical behavioral revealed here is not simply implied by the power-law distributed avalanche sizes. This can be demonstrated by studying the probability *p*_0_ of the quiescent state, i.e., all sites are inactive. This probability is not constrained by the power-law distribution in avalanche patterns (because it leads to divergence for a power-law), but nevertheless is important in order to obtain proper scaling and collapse using FSS. In the original data, *p*_0_ decreased in a unique way with increase in system size *n* (Figure 11). When *p*_0_ was changed randomly with n, the functions could not be collapsed anymore despite the preservation of the power-law in size distribution (Figure 12). Furthermore, we know that a system is not required to have power-law distributed avalanche sizes in order to exhibit features of equilibrium criticality. For example, Macke et al. (2011) has shown that for a system with (1) higher order interactions and (2) infinite correlation length, thermodynamic criticality is ensured, regardless of the pattern size distribution. Although the power-law size distribution is not necessarily associated with thermodynamic criticality, by testing a wide range of *T*, we found that the particular value of *T* that minimizes the distance from a power-law and the actual distribution is very close to 1 (0.99 ± 0.03; mean ± SD across eight sub-groups from 2 monkeys for the best fitting power-law and 1.03 ± 0.10 for the power-law with slope −1.5; Figure 13), demonstrating that there is a unique “temperature” associated with the avalanche dynamics. Given that there is no trivial relation between the power-law size distribution and the thermodynamic criticality, our finding that cortical dynamics exhibit these two features simultaneously is intriguing.

## Discussion

Our results suggest that neuronal avalanches at both mesoscopic and macroscopic scales manifest a cortical state near thermodynamic criticality. The critical exponents found are highly consistent among different subjects and are reasonably consistent across the two different scales and species. Our results are reminiscent of the well-known fact that, near the critical state, emergent behaviors do not depend on the specific microscopic realization of a system and, therefore, a multitude of systems can be categorized into a small number of universality classes based on their critical exponents (Stanley, 1987, 1999; Binney et al., 1992; Sornette, 2006). Our results thus suggest a general principle governing the collective behavior of cortical activities across spatial scales.

### Methodological considerations

**Figure A2:**
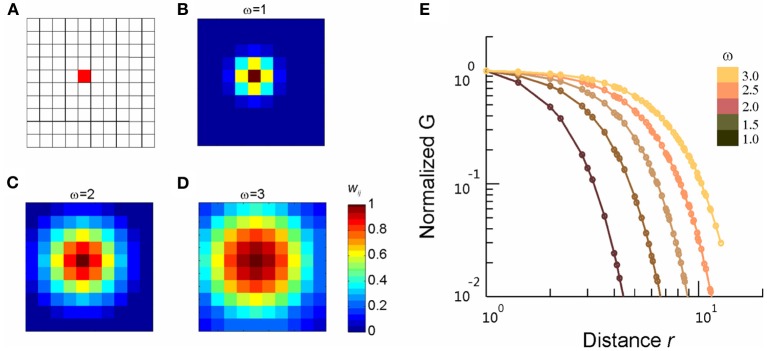
**Volume conduction does not introduce long-range correlations.** In a simulated 10 × 10 array configuration, we introduce volume conduction by mixing the independent signals from nearby sites according to *S*′_*i*_ = ∑ w_*ij*_S_*j*_, where *S* represents independent signals from individual site and *S'* represents the signals after mixing. The mixing weight $w_{ij}=\exp\left[-\frac{1}{2}{\left(\frac{r_{ij}}{\omega }\right)}^{2}\right]$, where *r*_*ij*_ is the distance between site *i* and *j*. **(A)** Spatial configuration of the simulated array. **(B–D)**, For an example site (the red square in **A**), the mixing weight *w*_*ij*_ (color corded) with different spatial extend (ω) are shown, with the reference to the whole array. **(E)** Correlation, *G*, between mixed activities are plotted as a function of the separation distance for different ω (color-coded). To facilitate comparison, *G* is normalized by the correlation between the nearest neighbors, i.e., *r* = 1.

We demonstrated previously that the nLFP correlates with local neuronal synchrony and increased spiking activity from local neuronal populations (Petermann et al., 2009; Yu et al., 2011). However, the exact spatial extent of the LFP is still debated. While some studies suggest that the LFP reflects neuronal activities within the vicinity of the microelectrode (<0.2 – 0.4 mm radius; Katzner et al., 2009; Xing et al., 2009), some evidence has been provided that even distant (>1 mm) neuronal activities might contribute to the LFP due to volume conduction (e.g., Kajikawa and Schroeder, 2011). Similar concerns are also related to MEG signals, as one sensor of the MEG can detect signals generated by multiple sources. A question thus arises as to what extent linear mixing of signals from different sources might affect the results presented in the current study? In general, volume conduction and/or signal mixing cannot produce genuine critical behavior. Criticality relies on long-range correlations that emerge from cascades of local interactions. That is, the activity of unit *A* affects unit *B*, which in turn affects unit *C*, and so on. As a result, the activity of unit *A* will be correlated (with some temporal delay) with a distant unit *X* (Stanley, 1999). If measured interactions solely arose from volume conduction and/or signal mixing, the activity of a local unit will not causally affect nearby units and, therefore, causal chains of interactions cannot form. Accordingly, volume conduction and/or linear signal mixing should not lead to the appearance of critical dynamics. We verified this statement by modeling volume conduction in a 10 × 10 array configuration, in which even fairly strong volume conduction fails to reproduce long-range correlations as observed in our neuronal data (see Appendix Figure A2). Furthermore, the FSS method we used here to identify criticality is robust to a potential contribution from volume conduction. This can be easily seen in the ring model we used to identify scaling collapse. Introducing volume conduction into the ring model is equivalent to an increase in ω, which controls the spatial extent of covariance between nearby elements. Our simulations demonstrated that even strong volume conduction (ω = 5) failed to produce the critical behavior as observed in our neuronal data (cf. Figure 8). These analyses suggest that our conclusions are unlikely to be affected by volume conduction or signal mixing.

A recent study (Mastromatteo and Marsili, 2011) reported that experimental data might falsely imply criticality due to (1) the limitation of finite sampling and (2) the bias introduced when choosing parameters to achieve best accuracy in the inferring procedure. However, neither aspect applies to the current study. The pair-wise correlation we observed for nLFPs that constitute neuronal avalanches are within the range of 0.2 – 0.6 (Pearson's *r*) and, given our sample sizes, the margin of error is <0.05 (95% confidence interval). Therefore, our sample sizes were large enough to infer even lower or higher correlation strengths [indicating larger distances from the critical state, see Mastromatteo and Marsili (2011)], if they actually existed in the system. This suggests that the proximity to a critical state is a true feature of the cortex. Furthermore, in the current analysis, no parameter for analyzing the data was chosen according to the criterion of inferring accuracy. Taken together, the current results are robust, in light of the known methodological biases.

### Suggestions of a new universality class for the resting brain

One of the key steps in our analysis was the use of the single histogram method to infer system behavior for different values of the control parameter *T*. This is a well-established method and has been widely applied to study various empirical systems and models at, or close to equilibrium (Tkacik et al., 2009; Macke et al., 2011; Stephens et al., 2013). Using the same method, Stephens et al. (2013) recently found that the spatial pattern of natural images contains indications of criticality. Macke et al. (2011) found that if a system exhibits higher-order interactions, its specific heat will diverge as long as the correlation does not decay as a function of the distance. In a study of spiking activities in salamander retina (Tkacik et al., 2009), it was found that the maximal heat capacity increased with system size and the corresponding *T* (*T*_peak_) approaches 1. This was suggested as evidence for criticality (Tkacik et al., 2009). Heat capacity, though, is an extensive quantity and thus, an increase in heat capacity with increasing system size is difficult to interpret. It does not necessarily indicate an increase in specific i.e., normalized, heat capacity. Furthermore, without a sound extrapolation of *T*_peak_ for *n* → ∞, it is difficult to give an accurate estimation of *T*_*c*_. In the current study, we took several steps to avoid such ambiguities. First, specific heat *C* was analyzed directly. More importantly, we used FSS to estimate both *T*_*c*_ and the critical exponents, providing a quantitative characterization of the system's behavior.

Interestingly, the critical exponents derived for the cortical activities are different from those that are commonly found in physics such as the Ising model, Heisenberg model or Spherical model (Binney et al., 1992). Cortical activity has distinctive features, including a currently unknown dimensionality and a special structure of higher-order interactions (Yu et al., 2011), which may underlie its unique critical exponents. We also notice that the value of β is close to zero, which in some cases indicates that the phase transition is a discontinuous one (Achlioptas et al., 2009). However, recently it was found that some continuous phase transitions have β so close to zero that it is practically indistinguishable from a discontinuous one (Riordan and Warnke, 2011). To further elucidate this issue, future work with approaches that can analyze much larger systems, i.e., larger *n*, would be needed to increase the precision in estimating *T*_*c*_ and critical exponents.

### Non-equilibrium and equilibrium perspectives of neuronal avalanche dynamics

Our current approach did not address the organization of activities within individual avalanches. It has been previously demonstrated that such activities can be effectively understood in the framework of a critical branching process (Beggs and Plenz, 2003; Shew et al., 2009, 2011; Friedman et al., 2012; Yang et al., 2012). That approach considers the spatiotemporal organization of events (nLFPs) that occur in an avalanche to be the result of balanced cascades and correctly predicts the power-law distribution in avalanche size with the exponent of –1.5. The critical branching process is a well-studied, non-equilibrium critical condition, which belongs to the universality class of directed percolation (Buice and Cowan, 2007). By collapsing the temporal dimension, we compressed the spatiotemporal pattern of neuronal cascades into spatial-only patterns and thus ignored the non-equilibrium cascading process in our present study. At the same time, we analyzed the ensemble of all cascades as a whole. Thus, our approach focused on the organization of avalanche activities at a different level. With this regard, the current results provide a complementary view to better understand cortical dynamics, suggesting a highly organized, hierarchical organization of cortical activity. We propose that cortical dynamics are organized close to criticality from both the non-equilibrium, branching process perspective and the equilibrium thermodynamic perspective. The former is indicated by a power-law size distribution, whereas the latter is indicated by *T*_*c*_ close to 1. Interestingly, recent studies that investigated large scale (across the entire brain) neuronal dynamics have also reported evidence for criticality in an equilibrium as well as non-equilibrium context (Deco and Jirsa, 2012; Haimovici et al., 2013; Shriki et al., 2013). Future studies to investigate how the brain can achieve both types of criticality, at different spatial as well as temporal scales hold great promise to uncover a more complete picture of cortical dynamics.

For the non-equilibrium critical state characterized by power-law probability distributions, theoretical as well as empirical studies have revealed functional advantages for neuronal information processing (Kinouchi and Copelli, 2006; Rämö et al., 2007; Shew et al., 2009, 2011; Tsubo et al., 2012; Yang et al., 2012). The equilibrium, thermodynamic criticality also has direct functional implications. From an information-theoretical point of view, the maximal specific heat, i.e., maximal variance of log(*p*_*i*_), implies largest dynamic range for population coding (Tkacik et al., 2009; Macke et al., 2011). This is also consistent with the finding that the dynamics of the brain reach highest signal complexity near the equilibrium criticality (Deco and Jirsa, 2012). The maximal susceptibility has an even more straightforward interpretation: it means that cortical networks have obtained largest sensitivity to small perturbations. This may play an essential role in allowing the organism to be able to detect and respond to subtle environmental changes. Such a high sensitivity of cortical networks has been demonstrated empirically for both spiking activity (Houweling and Brecht, 2007; Huber et al., 2008) and neuronal population activity reflected in the LFPs (Shew et al., 2009). The current results provide new insights into these intriguing phenomena of cortical dynamics.

### Potential functional role of the control parameter T in the brain

In systems studied in statistical mechanics, increasing the temperature *T* drives the system toward a state of higher activity and weaker effective interactions among the system components. Similar changes in activity and interactions have also been observed in the brain, specifically the cortex. For example, an increase in firing rate that is accompanied by a decrease in pair-wise correlation has been documented in transitions from a less vigilant state to a more vigilant state, e.g., from sleep to wakefulness (Vyazovskiy et al., 2009; Grosmark et al., 2012) and from an inattentive to an attentive state (Cohen and Maunsell, 2009; Harris and Thiele, 2011; Mitchell et al., 2009). These observations suggest that there might be intrinsic neural mechanisms for adjusting cortical states roughly along the same dimension as changing *T*.

It is well-known that neuromodulators, such as acetylcholine (ACh) and dopamine (DA) produce numerous diverse effects at the receptor, synaptic transmission, and single neuron level (Picciotto et al., 2012; Tritsch and Sabatini, 2012). On the other hand, when studying the effect of e.g., ACh in the context of cortical state changes (Himmelheber et al., 2000; Jones, 2005; Brown et al., 2011), effects brought about by an increase in the tone of ACh are quite reminiscent of the effects of increasing *T* in our framework. In particular, ACh drives cortical networks toward a state of high activity and weak coupling both *in vitro* (Chiappalone et al., 2007; Pasquale et al., 2008) and *in vivo* (Goard and Dan, 2009; Thiele et al., 2012). Similarly, the neuromodulator dopamine was shown to control neuronal avalanche dynamics via an inverted-U profile typical for the regulation of working memory (Stewart and Plenz, 2006). At moderate dopamine D1-receptor stimulation, neuronal avalanche dynamics was established, whereas lower or higher receptor stimulation abolished avalanche dynamics and reduced the number of local synchronized events reminiscent of weaker coupling between neurons.

The control parameter *T* might not capture the effects of changing the balance of fast excitation to fast inhibition (E/I) in a network. Experimentally, it has been shown that a proper E/I balance is required to maintain avalanche dynamics in cortical networks (Beggs and Plenz, 2003; Shew et al., 2009, 2011; Yang et al., 2012). Neuronal simulations have demonstrated that such proper E/I balance, in addition, establishes long-range temporal correlations in the network (Poil et al., 2012) as identified in the human EEG (e.g., Linkenkaer-Hansen et al., 2005; Montez et al., 2009). An increase in excitation, e.g., by reducing inhibition, increases activity. However, it also leads to an increase, not a decrease, in coupling (Shew et al., 2009, 2011).

## Concluding remarks

By studying neuronal avalanches in non-human primates and human subjects, we demonstrated that ongoing resting activity in the cortex organizes close to a thermodynamic critical point. We derived the cortical equivalents of the three parameters, including susceptibility, specific heat capacity and an order parameter that are commonly used in statistical mechanics to capture the behavior of systems near a thermodynamic critical point. By investigating the scaling behavior of these parameters we uncovered a potentially new universality class for the brain and propose that this endows cortical networks with maximized input sensitivity and dynamic range for representing information. Our results reveal, in a quantitative manner, how the interactions among individual neurons in cortex collectively give rise to emergent behavior that is highly non-trivial. With ever increasing capacity of monitoring activities of large neuronal networks, we anticipate that the framework provided here will be instrumental for understanding how cortical states are regulated through myriads of neuronal interactions to optimize information processing.

## Author contributions

Conceived and designed the experiments: Shan Yu, Hongdian Yang, Oren Shriki, and Dietmar Plenz. Performed the experiments: Shan Yu and Oren Shriki. Analyzed the data: Shan Yu, Hongdian Yang, and Oren Shriki. Contributed reagents/materials/analysis tools: Hongdian Yang. Wrote the paper: Shan Yu, Hongdian Yang, Oren Shriki, and Dietmar Plenz.

### Conflict of interest statement

The authors declare that the research was conducted in the absence of any commercial or financial relationships that could be construed as a potential conflict of interest.

## Appendix

### A. Examining the assumptions about stationarity and equilibrium

Thermodynamic equilibrium implies that the macroscopic properties of the system keep stable and do not change with time. As the distribution of avalanche sizes captures the essential feature of cortical dynamics (Beggs and Plenz, 2003; Petermann et al., 2009; Shew et al., 2009, 2011; Yang et al., 2012), we examined the stability of this size distribution. In Figure 3 (main text), we show that the avalanche size distribution, measured for 10 consecutive, equal-sized segments of recording, is stable across the whole recording period (30 min). To contrast this with the true equilibrium condition, we shuffled the original avalanche raster (i.e., randomized its sequence) and repeated the same analysis. The variability of the estimated power law exponent, ε, across all segments is small for both the original and shuffled datasets. *F*-test statistics also revealed no significant difference in the variance of ε between the two conditions (*p* = 0.13), suggesting a stable organization of the system over time.

Secondly, we demonstrate that the data satisfy two crucial criteria that will lead to equilibrium: (1) detailed balance (micro-reversibility) and (2) accessibility/ergodicity (Binney et al., 1992). Detailed balance is achieved in a system if the following relation holds: *p*_*i*_*p*_*i*→*j*_ = *p*_*j*_*p*_*j*→*i*_, where *i* and *j* are possible states (configurations) of the system; *pi* is the probability of states *i* and *p*_*i*→*j*_ is the transition probability from state i to state j. For avalanche patterns defined by clustering a period of activity flanked by quiescent periods before and after it (Beggs and Plenz, 2003), it is clear that the detailed balance strictly holds for systems with arbitrary sizes. As in this condition, every transition from a quiescent state (i.e., all sites are inactive) to an active state (i.e., at least one of the sites is active) would be accompanied by a reverse of that transition. In other words, the system will satisfy *p*_*q*_*p*_*q*→*i*_ = *p*_*i*_*p*_*i*→*q*_, where *q* is the quiescent state and *i* is any active state. Such a feature, combined with the fact that all *p*_*i*→*j*_ = 0 when *i* and *j* are both active states, ensures the detailed balance.

To study whether the detailed balance still holds when we release these constraints set by the rules that identify avalanches, we examined the relation between *p*_*i*_*p*_*i*→*j*_ = *p*_*j*_*p*_*j*→*i*_ in the data with quiescent periods removed. In such case, both constraints, i.e., the symmetrical transition from a quiescent state to an active state and zero transition probability between active states, are removed. In Figure A1, we plotted the measured *p*_*i*_*p*_*i*→*j*_ against *p*_*j*_*p*_*j*→*i*_ for systems with different sizes (*n* = 2–5). Overall, the data points are fairly close to the identical line, suggesting the fulfillment of the equality. For comparison, we constructed a shuffled data set, in which the sequence of avalanche patterns was randomized. For this shuffled data set, any possible temporal dependency was removed so it is in a truly equilibrium state and, therefore, fulfills the detailed balance. The same analysis was then performed for the shuffled data and we found that the results are similar to those from the original data, indicating that the deviation from the identical line for the original data is largely due to finite sampling, and not due to a violation of the detailed balance. To quantify this effect, we computed the ratio *r* = *D*_data_/*D*_shuffled_, where

(A1) $$D=\frac{2\left|p_{i}p_{i\to j}-p_{j}p_{j\to i}\right|}{p_{i}p_{i\to j}+p_{j}p_{j\to i}}$$

We found that for *n* > 2, this ratio is very close to one (Mann–Whitney U test, *p* > 0.05), indicating that the violation to the detailed balance is sufficiently small so it is not detectable within the current recording length. Due to the lack of sufficient data, the direct check of detailed balance cannot be performed for larger systems (*n* >> 5). However, with the results we obtained for *n* = 2–5, and given the fact that with the increase of system size, exponentially more samples would be needed to detect the same level of violation, it is clear that the detailed balance among the active states, i.e., avalanche patterns, should be a good approximation for even larger systems.

Regarding the accessibility/ergodicity assumption, it requires that from any given state, the system should be able to evolve (after a sufficiently long time) to any other state. Although the direct test for ergodicity is not possible due to limited length of the recording, the power-law distribution in avalanche sizes provides strong empirical evidence to support it. Such a heavy-tailed distribution indicates that even large systems can visit configurations that cover all possible avalanche sizes.

Taken together, various empirical tests strongly suggest that the stationarity and even the equilibrium assumption can be considered a reasonable first approximation for our data.

### B. Analytical derivation of finite size scaling method

For readers who are not familiar with the finite size scaling, we illustrate the method using susceptibility χ as an example. In the vicinity of the critical temperature *T*_*c*_, χ can be expressed as a function of correlation length ξ.

(A2) $$\chi =\xi^{\gamma /v}$$

In finite size system, correlation length ξ is comparable to system size *L*, and therefore has a cut off. Consequently, χ also has a cut off. If we use ξ to represent the correlation length at the thermodynamic limit, then the cut off takes place when ξ > L. Then, we can rewrite χ as

(A3) $$\chi =\xi^{\gamma /v}\chi_{0}(L/\xi ),$$

which satisfies the conditions above. Then define

(A4) $$\begin{array}{l} \chi_{0}(x)\sim \chi^{\gamma /v},\text{ for }x<1\\ \chi_{0}(x)\sim c,\text{ otherwise},\text{ where }c\text{ is a constant}. \end{array}$$

Therefore, when the system size is finite,

(A5) $$\chi_{L}=\xi^{\gamma /v}{\left(L/\xi \right)}^{\gamma /v}=L^{\gamma /v},$$

And the correlation length is comparable to the system size. Otherwise, when the system size is infinite, the correlation length is actually ξ,

(A6) $$\chi =c\xi^{\gamma /v}.$$

Now we can rewrite the equation in order to remove ξ, because we do not know its exact value, and also to introduce a dimensionless function $\bar{\chi }(x)$, which will be the scaling function for χ_*L*_

(A7) $$\begin{array}{l} \chi =\xi^{\gamma /v}\chi_{0}\left(L{\left|t\right|}^{v}\right)\\ \;=\xi^{\gamma /v}\chi_{0}\left[{\left(L^{1/v}\left|t\right|\right)}^{v}\right]\\ \;=|t{|}^{-\gamma }\chi_{0}\left[{\left(L^{1/v}\left|t\right|\right)}^{v}\right]\\ \;=L^{\gamma /v}L^{-\gamma /v}{\left|t\right|}^{-\gamma }\chi_{0}\left[{\left(L^{1/v}\left|t\right|\right)}^{v}\right]\\ \;=L^{\gamma /v}{\left(L^{1/v}\left|t\right|\right)}^{-\gamma }\chi_{0}\left[{\left(L^{1/v}\left|t\right|\right)}^{v}\right]. \end{array}$$

Set *x* = *L*^1/*v*^|*t*|, which will be the scaling variable

(A8) $$\chi =L^{\gamma /v}x^{-\gamma }\chi_{0}(x^{v})$$

Define scaling function $\bar{\chi }(x)=x^{-\gamma }\chi_{0}(x^{v})$, then

(A9) $$\chi =L^{\gamma /v}\;\bar{\chi }(L^{1/v}\left|t\right|).$$

Note when ξ ~ *L*,

(A10) $$\begin{array}{l} \bar{\chi }(x)=x^{-\gamma }\chi_{0}(x^{v})\\ \;={\left(L^{1/v}\left|t\right|\right)}^{-\gamma }\chi_{0}\left[{\left(L^{1/v}\left|t\right|\right)}^{v}\right]\\ \;=L^{-\gamma /v}{\left|t\right|}^{-\gamma }\chi_{0}\left(L{\left|t\right|}^{v}\right)\\ \;=L^{-\gamma /v}{\left|t\right|}^{-\gamma }c{\left(L{\left|t\right|}^{v}\right)}^{\gamma /v}\\ \;=L^{-\gamma /v}{\left|t\right|}^{-\gamma }cL^{\gamma /v}{\left|t\right|}^{\gamma }\\ \;=c \end{array}$$

Thus, the scaling function is a constant and independent of the system size.

The scaling function also can be written as

(A11) $$\begin{array}{l} \bar{\chi }\left(L^{1/v}|t|\right)=L^{-\gamma /v}|t{|}^{-\gamma }c{\left(L|t{|}^{v}\right)}^{\gamma /v}\\ \;=L^{-\gamma /v}{\left(|t{|}^{-\gamma }\right)}^{\gamma /v}c{\left(\frac{L}{|t{|}^{-v}}\right)}^{\gamma /v} \end{array}$$

Recall ξ ~ |*t*|^−*v*^, so we have

(A12) $$\bar{\chi }\left(L^{1/v}|t|\right)=L^{-\gamma \text{/}v}\;\xi^{\gamma /v}c\left(\frac{L}{\xi }\right){\text{​}}^{\gamma /v}$$

Also recall, when system size is finite,

(A13) $$\chi_{L}=\xi^{\gamma /v}{\left(L/\xi \right)}^{\gamma /v},$$

So

(A14) $$\bar{\chi }\left(L^{1\text{/}v}|t|\right)=L^{-\gamma \text{/}v}\chi_{\text{L}}=c$$

From Eq. A14, we can measureχ_*L*_(*t*) for various system sizes *L* in a temperature range close to *T*_*c*_, and rescale χ_*L*_(*t*) by *L*^−γ/*v*^ for each L to obtain the scaling function$\bar{\chi }(L^{1/v}|t|)$, with *L*^1/*v*^|*t*| as the scaling variable. If we choose the correct *T*_*c*_, ν and γ, the scaling functions for different system sizes will fall on the same curve.
